# Fluorescent transgenic mouse models for whole-brain imaging in health and disease

**DOI:** 10.3389/fnmol.2022.958222

**Published:** 2022-09-23

**Authors:** Adrian Arias, Linus Manubens-Gil, Mara Dierssen

**Affiliations:** ^1^Department of System Biology, Centre for Genomic Regulation, The Barcelona Institute of Science and Technology, Barcelona, Spain; ^2^Institute for Brain and Intelligence, Southeast University, Nanjing, China; ^3^Department of Experimental and Health Sciences, University Pompeu Fabra, Barcelona, Spain; ^4^Centro de Investigación Biomédica en Red de Enfermedades Raras (CIBERER), Barcelona, Spain

**Keywords:** fluorescent transgenic models, whole-brain imaging, cell detection and counting, morphological reconstruction, computational neuroscience

## Abstract

A paradigm shift is occurring in neuroscience and in general in life sciences converting biomedical research from a descriptive discipline into a quantitative, predictive, actionable science. Living systems are becoming amenable to quantitative description, with profound consequences for our ability to predict biological phenomena. New experimental tools such as tissue clearing, whole-brain imaging, and genetic engineering technologies have opened the opportunity to embrace this new paradigm, allowing to extract anatomical features such as cell number, their full morphology, and even their structural connectivity. These tools will also allow the exploration of new features such as their geometrical arrangement, within and across brain regions. This would be especially important to better characterize brain function and pathological alterations in neurological, neurodevelopmental, and neurodegenerative disorders. New animal models for mapping fluorescent protein-expressing neurons and axon pathways in adult mice are key to this aim. As a result of both developments, relevant cell populations with endogenous fluorescence signals can be comprehensively and quantitatively mapped to whole-brain images acquired at submicron resolution. However, they present intrinsic limitations: weak fluorescent signals, unequal signal strength across the same cell type, lack of specificity of fluorescent labels, overlapping signals in cell types with dense labeling, or undetectable signal at distal parts of the neurons, among others. In this review, we discuss the recent advances in the development of fluorescent transgenic mouse models that overcome to some extent the technical and conceptual limitations and tradeoffs between different strategies. We also discuss the potential use of these strains for understanding disease.

## Introduction

Investigating the nervous system from the perspective of the neuron doctrine has provided neuroscientists with ever-growing details of the anatomy of single neurons and the composition of neuronal circuits. The original developments of the Golgi’s method and its variants, with random sampling of sparse cells, already allowed to grasp fundamental aspects of the system (Ramon and Cajal, 1904). The advent in the last decade of brain clearing techniques, fluorescent cell labeling, and whole-brain imaging based on optical (e.g., light-sheet fluorescence microscopy; LSFM) and automated mechanical sectioning (e.g., fluorescence micro-optical sectioning tomography; fMOST), along with new data analysis and management methods, is transforming the central nervous system into a structure amenable to quantitative description, with profound consequences on our ability to predict biological phenomena. Embracing this new paradigm provides an excellent opportunity to precisely quantify numbers of cells, or study the relevance of fine structural details in neurons, and their impact on mesoscopic neural networks, and ultimately on the whole brain. Having such a holistic view is converting neuroscience research from a descriptive discipline into a quantitative, predictive, actionable science that may ultimately reveal the mechanism by which specific architectural alterations correlate with cognitive performance. These advancements also hold the promise to reframe our understanding of neuropathology, thus leading to new hypotheses and unexplored pathways to therapy.

**FIGURE 1:**
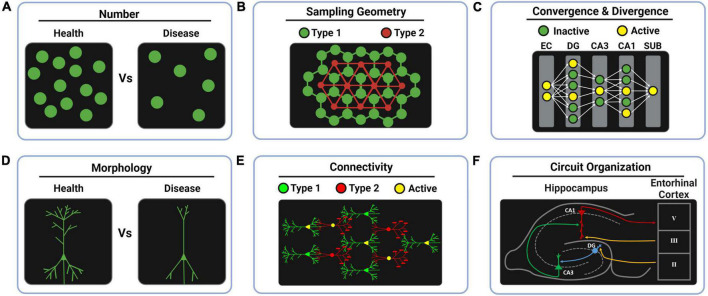
Whole-brain anatomical features for computational neuroscience. **(A)** The computational redundancy of the brain can be studied by cell counting in health and disease to predict the minimal percentage of neuronal loss that leads to symptoms. **(B)** Studying the sampling patterns that the brain uses for covering the space can reveal different design principles across brain regions such as the regular mosaic principle found in tagged functional cell types of the retina. In the drawings, lines between cells represent spatial distances, not real connections. **(C)** Encoding transformations can be interrogated by studying increments (divergence) or decrements (convergence) in neuron number across layers. Likewise, sparse coding and dense coding strategies might be revealed by tagging the percentage of active neurons in each layer during behavioral tasks. **(D)** The computational impact of morphological features can be studied by comparing health and disease models. **(E)** Data-driven morpho-neuronal models can be elaborated to understand the design principles behind structural connectivity. **(F)** Genetically labeling functional cell types provides a way to ultimately study the fine circuit organization of complex brain regions. EC, entorhinal cortex; DG, dentate gyrus; CA3, Ammon’s horn field 3; CA1, Ammon’s horn field 1; SUB, subiculum.

An important aspect of all these new approaches is the need of specific animal models for mapping fluorescent protein-expressing neurons for visualizing their mesoscopic anatomical features and their impact on brain computation (see Figure 1). This view was not totally addressed by previous reviews on fluorescent transgenic mouse models which were mainly focused on molecular neurobiology (Hadjantonakis et al., 2003; Nowotschin et al., 2009; Taraska and Zagotta, 2010; Abe and Fujimori, 2013; Enterina et al., 2015; Navabpour et al., 2020), and only a few of them approach a computational neuroscience perspective (Josh Huang and Zeng, 2013; Soden et al., 2014).

## Whole-brain fluorescent imaging

The development of organic solvent-based (BABB; Dodt et al., 2007 and 3DISCO; Ertürk et al., 2012), aqueous-based (Scale; Hama et al., 2011 and SeeDB; Ke et al., 2013), and hydrogel embedding (CLARITY; Chung et al., 2013) tissue clearing techniques kickstarted the emergence of several method variants for whole-organ imaging (Tian et al., 2021). Those, combined with the fast development of light-sheet fluorescence microscopy (LSFM; Verveer et al., 2007) two-photon microscopy (Lidke and Lidke, 2012) or stimulated Raman scattering microscopy (Wei et al., 2019), have allowed imaging of biological samples at the scale of cm^3^ with micrometric resolution in few hours (Tomer et al., 2014; Wei et al., 2019) and recently improved to minutes-timescale acquisition (Fang et al., 2021). When fluorescent labeling is bright and specific enough, those techniques allow cellular resolution structural phenotyping of full rodent brains (Mano et al., 2018; Ueda et al., 2020). While several efforts focused on improving the speed and imaging quality of cleared brain samples (Richardson and Lichtman, 2015; Yu et al., 2018; Weiss et al., 2021), others have focused on reaching homogeneous immunostaining (Renier et al., 2014; Park et al., 2019). Automated mechanical sectioning of tissue samples has been used as an alternative to tissue clearing techniques, allowing to circumvent the technical issues associated with intact sample imaging and allowing to take advantage of well-optimized confocal imaging methods. Serial two-photon tomography (STP; Amato et al., 2016), block-face serial microscopy tomography (FAST; Seiriki et al., 2017), and fMOST (Zheng et al., 2013) have been used thoroughly for studying the anatomy of whole rodent brains (Zheng et al., 2019). Simultaneous with the development of imaging technologies, the need for image processing tool sets for terabyte-sized datasets has emerged. The development of a common atlas of the mouse brain (Common Coordinate Framework v3; Wang et al., 2020), combined with image stitching (Bria and Iannello, 2012; Wang et al., 2020), transformation to multi-resolution image formats (Bria et al., 2016), and spatial registration (Tward et al., 2020; Chandrashekhar et al., 2021; Jin et al., 2022; Qu et al., 2022), allows the quantification of cell densities (Renier et al., 2016), axonal projections (Ye et al., 2016), vasculature (Kirst et al., 2020), and the reconstruction of full single neurons (Winnubst et al., 2019; Peng et al., 2021; Gao et al., 2022).

## Imaging fluorescent transgenic mice: Potential and limitations

**FIGURE 2:**
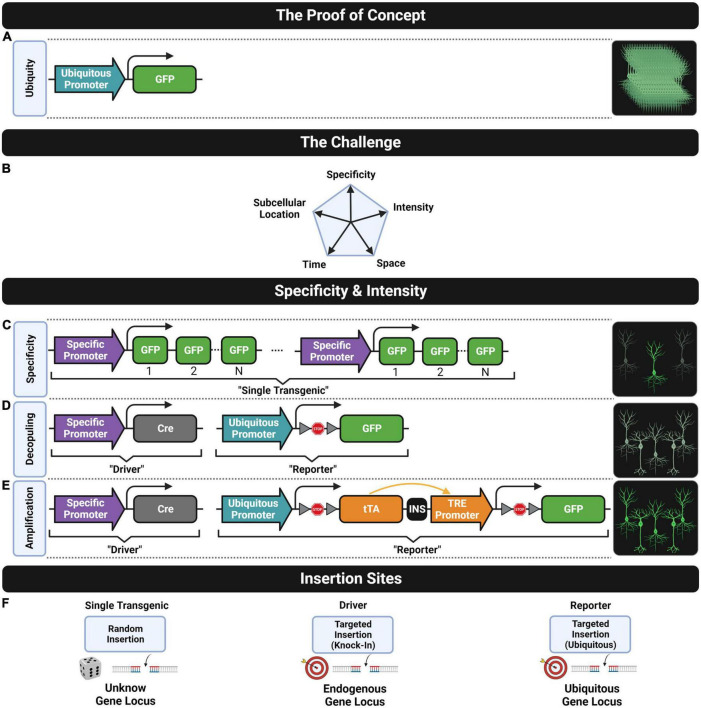
Fluorescent transgenic models. **(A)** The construct design that leads to the ubiquitous cell expression of the green FP as the proof-of-concept model known as the “green mice”. **(B)** The challenge of developing genetic tools for controlling five fundamental features of transgene expression for neuroscience research. Cell-type specificity can be achieved through **(C)** a single or **(D)** a double transgenic strategy. The intensity of the fluorescent signal can be increased through **(C)** including multiple transgene repeats, **(D)** using a stronger ubiquitous promoter or **(E)** amplifying the expression of a single transgene copy. **(F)** Common insertion sites of transgene constructs into the genome for generating lines following (left) the single or (middle and right) the double transgenic approach.

In the simplest design of a fluorescent transgenic model, the selected promoter that drives the expression of the fluorescent protein (FP) commonly determines the targeted cell subtype to be labeled (specificity), the expression levels of the transgene (intensity), the distribution of labeled cells (space), and the expression regulation and turnover (time). In addition, the FP is by default expressed in the cytosol, where its specific distribution (subcellular location) is determined by free diffusion (Van Den Pol and Ghosh, 1998; Yamaguchi et al., 2000), but it can also accumulate in the nucleus forming bright speckles in some cells. The lack of control over these variables, which is depicted in Figure 2B, may constitute a technical limitation in many experimental applications. In the following sections, we review the implications of this default mode of expression of the transgene and new methods to address such uncontrolled expression. Moreover, the applicability of these advances to whole-brain analysis will also be discussed across sections.

## From ubiquitous to cell-specific genetic labeling

With the advent of single nuclei sequencing techniques, a myriad of new neuronal subtype classifications, defining the morphological and functional properties of particular cell types, has become increasingly complex (Zeng and Sanes, 2017). Thus, developing versatile mouse models able to label selectively defined cell types is more and more necessary. This was not possible with the first transgenic mouse models expressing FPs. At the end of the 20th century, a transgenic mouse expressing a fluorescent protein (enhanced GFP; eGFP) driven by a ubiquitous promoter (cytomegalovirus early enhancer element, chicken beta-actin promoter, and rabbit beta-Globin splice acceptor site; CAG) was generated (Okabe et al., 1997). With the exception of hair and blood red cells, the fluorescent signal is present in all cells of this mouse model and can be easily detected by the naked eye. However, the shapes of individual cells are impossible to determine, since the ubiquitous expression of fluorescent signals impedes resolving cell boundaries (Figure 2A).

Transgenic strategies using a cell-type-specific promoter sequence followed by multiple FP sequences (Figure 2C) allow labeling of particular cell subpopulations, such as hypothalamic neurosecretory cells with the gonadotropin-releasing hormone-GFP (GnRH-GFP) line (Spergel et al., 1999) or the melanocortin-4 receptor (MC4-R-GFP) line (Liu et al., 2003), pyramidal neurons using the Thy1 promoter and enhanced yellow fluorescent protein (Thy1-eYFP) line (Feng et al., 2000), pan-GABAergic neurons using the 67 kDa glutamic acid decarboxylase (GAD67)-eGFP line (Oliva et al., 2000), neural progenitor cells in the nestin-GFP line (Yamaguchi et al., 2000), Purkinje cells of the cerebellum in the calbindin (Calb)-GFP line (Maskos et al., 2002), fast-spiking interneurons of the cortex and hippocampus using the voltage-dependent potassium channel Kv3.1-eYFP line (Metzger et al., 2002), or cholinergic neurons in the choline acetyltransferase (ChAT)-eGFP line (Tallini et al., 2006), among many others.

In parallel, a double transgenic strategy was proposed for achieving cell-type specificity based on the Cre/LoxP recombination system (Sternberg and Hamilton, 1981; Sauer, 1987; Sauer and Henderson, 1988), as shown in Figure 2D. One transgenic line known as “the driver” is designed by targeting the Cre sequence into a specific gene locus (i.e., knock-in strategy, see middle drawing in Figure 2F) where Cre expression is driven by a cell-type-specific endogenous promoter under interest. The other transgenic line known as “the reporter” is designed by targeting to a ubiquitous gene locus (see left most drawing in Figure 2F), a construct consisting of a ubiquitous promoter (e.g., CAG) followed by a LoxP-STOP-LoxP cassette (LSL) preceding a fluorescent transgene. Crossing these two lines results in a double transgenic line where the STOP sequence is removed specifically in those cell types where Cre is expressed leading to the expression of the fluorescent protein. The first proof of concept of this design was done by crossing a ubiquitous Cre driver (CAG-Cre) with a fluorescence Cre-inducible reporter line (CAG-LSL-eGFP) resulting in a double transgenic with whole-body fluorescence (Kawamoto et al., 2000). Then, cell-specific labeling was further achieved by crossing cell-type-specific Cre drivers with fluorescent Cre-inducible reporters, for example, to express fluorescence in epidermal cells with the bovine keratin 5 (KRT5) K5-Cre;CAG-LSL-eGFP line (Kawamoto et al., 2000), or in early embryogenesis cells with the binding protein GATA1 promoter combined with the ubiquitous promoter reverse orientation splice acceptor (ROSA)26 in GATA1-Cre; ROSA26-LSL-eGFP (Mao et al., 2001), or in motor neurons Islet 1 sl1-Cre; ROSA26-eGFP (Srinivas et al., 2001), among others. Large-scale projects have generated multiple Cre mouse lines driven by different promoters for neuroscience research, based on the gene expression mapping in the mouse brain. Gene expression data can be found in repositories such as the Mouse Genome Informatics (MGI) database (Blake et al., 2003), the Gene Expression Nervous System Atlas (GENSAT) database (Heintz, 2004), or the Allen Institute Gene Expression Atlas of the Adult Mouse Brain (Lein et al., 2007). In fact, the Allen Institute’s Transgenic Characterization database (Harris et al., 2014) contains over 100 Cre driver lines developed by the institution, and extended lists of transgenic mouse databases can be found in several reviews (Anagnostopoulos and Eppig, 2014; Tsien, 2016; Clark et al., 2020). Currently, only the Allen Institute’s Transgenic Characterization database provides a description of some of the available fluorescent Cre-inducible reporters. However, a complete and specialized database to guide researchers to select the most suitable reporter line for their application is missing. These transgenic mouse drivers and reporters can be acquired through the principal providers such as Jackson^1^, MMRRC^2^, and BACPAC.^3^ In addition to the popular Cre/LoxP, there are other driver designs based on different site-specific recombinase (SSR) systems, such as the Flp/Frt (Andrews et al., 1985), Dre/Rox (Sauer and McDermott, 2004), or the more recent Nigri/Nox (Karimova et al., 2016), among many others (Tian and Zhou, 2021).

## Intensity of transgene expression

Labeling neurons with fluorescent signals that are bright enough to allow efficient imaging is fundamental for extracting accurate anatomical features. In some transgenic models, one can encounter suboptimal fluorescence conditions. For instance, when the soma is not bright enough in comparison with the surrounding background signal, random fluorescence patterns resembling a soma might be wrongly detected as neurons (false positive) while dim somas might be not detected (false negatives), and we cannot rely on automatic cell detection algorithms. Likewise, the fluorescent signal may not be equally distributed within the neuron. The signal tends to be brighter in coarse compartments, such as the soma, and dimmer in fine structures such as distal neurites (Schmidt et al., 2013). As a result, semiautomatic reconstruction algorithms tend to fail for accurate cell counting but also when tracking the full morphology, implying the need for manual proofreading by trained annotators.

There are many factors that can lead to a weak fluorescent signal, especially in those transgenic designs where a cell-type-specific promoter directly drives the expression of the fluorescent protein as is shown in Figure 2B. First, cell-type-specific promoters are unequally efficient in providing strong levels of fluorescent signal and usually have an incomplete promoter sequence that lacks upstream regulatory elements, such as gene expression enhancers, which also contributes to a weak expression. It is difficult to include these missing regulatory sequences into the design of the transgene since the length of the sequence that can be inserted into the genome is limited. The number of copies of the fluorescent transgene sequence that can be inserted in a suitable genome locus for gene expression is also a key factor that determines the strength of the fluorescent signal.

In fact, one of the first strategies to generate transgenic lines with brighter fluorescent signals consisted of increasing the number of transgene copies. Paradoxically, regions of the genome with repetitive gene sequences are more likely to lead to a reduced expression or even complete silencing of that gene in some cells (Henikoff, 1998; Akitake et al., 2011; Wei et al., 2015). As a result, multiple transgene repeats can lead to inconsistent labeling patterns (Feng et al., 2000; Josh Huang and Zeng, 2013) with huge differences in brightness across cells and missing unlabeled cells. A paradigmatic example of bright fluorescent labeling which is based on this strategy is the Thy1-eYFP-H line (Feng et al., 2000) where the Thy1 promoter drives a strong expression of the fluorescent protein mainly in pyramidal neurons (Caroni, 1997; Feng et al., 2000; Porrero et al., 2010; Heimer-Mcginn and Young, 2011). Besides the silencing events caused by multiple transgene repeats, in this line, the construct sequence is randomly inserted in loci of the genome (see left most drawing in Figure 2F) where transgene expression might not be guaranteed across cells, due to epigenetic factors. Therefore, the expression pattern in the Thy1-eYFP-H line is double biased by design, and only a small proportion of cells that express the Thy1 gene will be labeled.

Another drawback is that, depending on the background signal, the brightness can be strong enough or not for visualizing individual axons. For instance, there is at least one study using this transgenic line where reconstructions of individual axonal projections were possible due to the very low background (i.e., noise) signal of the imaging technique applied (Zhang et al., 2019) which requires of automated mechanical sectioning of resin-embedded whole brains. Moreover, the background signal can be even further reduced in this protocol by adding a light absorber to the resin (Gong et al., 2016; Zhang et al., 2019; Wang and Xiong, 2021). Instead, individual axons are not visible when cleared whole brains are scanned with optical sectioning techniques, such as light-sheet fluorescence microscopy (LSFM), which are characterized for having higher background signals (Qi et al., 2015; Zhang Z. et al., 2021). Thus, for those imaging techniques in which reducing the background signal is not possible, a brighter fluorescent signal such as the one achieved with adeno-associated virus infections might be required (Zhang Z. et al., 2021). Even having those limitations, these fluorescent transgenic lines based on multiple transgene repeats have been extensively used for many research applications and are still used nowadays. For instance, the Thy1-eYFP-H line was used to monitor the dendritic spine dynamics of pyramidal neurons in cortical layer 5 using *in vivo* transcranial two-photon microscopy in health (Grutzendler et al., 2002; Xu et al., 2007, 2009; Clark et al., 2018; Zhou et al., 2020) and disease (Pan et al., 2010; Bączyńska et al., 2021) for studying the plasticity of nervous systems during development stages, and learning and memory behavioral tasks (Pan and Gan, 2008; Fu and Zuo, 2011; Moyer and Zuo, 2018; Ma and Zuo, 2022), or dendritic stability of hippocampal pyramidal neurons in CA1 (Barretto et al., 2011). Moreover, the endogenous bright fluorescent labeling of these transgenic lines (mainly Thy1-eYFP-H and Thy1-eGFP-M lines) makes them suitable for being systematically used in mouse whole-brain imaging approaches as standards for validating many clearing (Chung et al., 2013; Susaki et al., 2014; Pan et al., 2016; Jing et al., 2018; Park et al., 2019) and imaging protocols (Gong et al., 2013, 2016; Xiong et al., 2014; Amato et al., 2016; Gang et al., 2017; Gao et al., 2019; Wang and Xiong, 2021; Zhang Z. et al., 2021).

Another strategy for increasing fluorescent intensity is generating double transgenic lines by crossing Cre-mediated drivers with Cre-inducible reporters. Four key design improvements were introduced by this approach that were relevant for mouse model reusability, brightness uniformity, and labeling completeness. First, the genetically defined cell specificity (driver line) is decoupled from the fluorescent transgene expression (reporter line, Figure 2C), as the same reporter line can be reused for labeling different cell types by just crossing it with other driver lines. Second, the expression of the fluorescent protein is driven by a strong ubiquitous promoter instead of a cell-specific one (compare Figures 2B,C). As a consequence, cells expressing low levels of the targeted endogenous gene will present similar brightness to those having higher endogenous gene expression. This results in a labeling pattern where brightness is more uniform allowing more consistent detection of complete cell populations. Of course, brightness cannot be used in this strategy as a putative readout of endogenous gene expression (Heintz, 2001; Schmidt et al., 2013). Third, the transgene construct is targeted to a permissive expression locus of the mouse genome (see the right most drawing in Figure 2F) instead of being inserted into a random locus (see the left most drawing in Figure 2F) which might not be epigenetically accessible in some intended cells. Fourth, only one single copy of the transgene is used to drive the expression instead of multiple ones thus reducing epigenetic silencing events. However, achieving high expression levels using only a single transgene copy is challenging. For this reason, the design of Cre-inducible reporter lines has been improved over the years to provide brighter fluorescent signals. Unfortunately, this usually came at the cost of introducing other undesired features.

The first Cre-inducible reporter designs used a ubiquitous promoter followed by a single copy of the fluorescent transgene (Figure 2C) usually targeted to the ROSA26 locus (Soriano, 1999), which provides ubiquitous expression (Zambrowicz et al., 1997). However, the expression of the first generation of these reporter lines was driven by pretty weak ubiquitous promoters such as phosphoglycerate kinase (pPGK) or pROSA26 (Mao et al., 2001; Srinivas et al., 2001) that lead to low intensity fluorescent signals. Subsequently, a brighter signal was reached by introducing two improvements: the use of another ubiquitous promoter (pCAG) that drives a stronger expression of the reporter and the inclusion of a posttranscriptional regulatory element of woodchuck hepatitis virus (WPRE) able to further enhance expression, *via* increasing the mRNA transcript stability (Zufferey et al., 1999). These improvements led to the first Cre-inducible reporter design (ROSA-CAG; producing the reporter mouse lines called Ai14 and Ai6, among others) able to reach a bright fluorescent signal by only using one copy of the fluorescence transgene instead of multiple ones (Madisen et al., 2010). Those lines were widely crossed with many Cre-mediated drivers to provide double transgenic lines for studying, for instance, the morphology of cortical chandelier cells with the Nkx2.1-CreER;Ai14 line (Ishino et al., 2017), the dopaminergic system with the D1-Cre;Ai6 and D2-Cre;Ai14 lines (Wei et al., 2018) and engrams formation with the Fos-CreER;Ai14 (Roy et al., 2022).

Some years later, a new Cre-inducible reporter design based on the Tet-On system (Zeng et al., 2008) was developed to provide a powerful transgene expression (Madisen et al., 2015; Daigle et al., 2018). The design is characterized for using the TRE promoter to amplify the expression of a single copy of the fluorescent transgene and for inserting the construct into a new tightly regulated genomic (TIGRE) locus that ensures no basal expression (Figure 2E). As a result, several TIGRE-TRE transgenic lines were developed (e.g., Ai82, Ai140, or Ai148, among others) to provide a strong gene expression comparable with those seen in adeno-associated virus (AAV) infections, allowing to resolve thinnest neuronal structures such as distal axons that were impossible to be visualized with reporter lines based on the ROSA-CAG design or multiple transgene repeats design, as the standard Thy1-eYFP-M line. Unfortunately, the bright fluorescent signal achieved with the TIGRE-TRE designs came at the cost of some adverse effects, such as aberrant morphologies in neurons, alterations in the dimensions of brain regions, embryonic lethality, or premature death (Daigle et al., 2018). These drawbacks are more likely observed when using Cre drivers with promoters that have a broad transgene expression in the brain and/or body such as the pan-neuronal Camk2a-Cre driver or the pan-cortical Emx1-Cre driver. However, embryonic lethality also was reported with the pan-GABAergic Gad2-Cre driver that is more sparsely expressed. The authors warn about the use of Cre driver lines with widespread and early developmental transgene expression. The use of CreER drivers (which will be described in detail in Section “Inducible Systems”) can solve in some cases these issues by switching on the expression of Cre-dependent reporter lines once development is finished in the adult mouse (Daigle et al., 2018). For instance, adverse effects were seen in the driver-reporter combination of Camk2a-Cre;Ai148 but not in the Camk2a-CreER;Ai148. Unfortunately, there is still no estimation of how long it takes, after tamoxifen activation, to reach the optimal fluorescent signal for imaging without causing any adverse effect in mice.

The intensity of the fluorescent signal can also be improved by crossing two Cre-inducible reporter lines so that two alleles express the fluorescent transgene. This approach was successfully applied for reconstructing the morphology of thalamic (Tnnt1-IRES2-CreERT2; Ai82; Ai140), cortical (Plxnd1-CreER; Ai82; Ai140), and claustral (Gnb4-IRES2-CreERT2; Ai140; Ai82) neurons (Wang et al., 2019; Peng et al., 2020, 2021).

## Spatial distribution of genetically defined subpopulations

Spatial sparsity is another interesting feature as it is essential to provide high-quality morphological reconstructions. Extracting anatomical features from a very dense spatial distribution of fluorescent cells is almost impossible and constitutes, in most cases, an unsolved challenge. This is especially true when using algorithms designed to automatically extract full morphological reconstructions of neurons. Although different possible solutions have been explored, most tend to fail when light signals of different neurons are too close or even overlapped, making it challenging to disentangle to which cell each light signal belongs (Li et al., 2019). In this regard, it is important to point out that the spatial distribution can be extremely dense when using fluorescent transgenic models driven by pan-neural promoters (e.g., Camk2 or Snap 25) or relatively sparse when using pan-GABAergic promoters (e.g., Slc32a1 or Gad67). For this reason, several strategies were assayed to develop fluorescent transgenic lines that label spatially sparse subsets of genetically defined cell types. However, sparsity is difficult to achieve without biasing the labeled subset, as discussed below.

**FIGURE 3:**
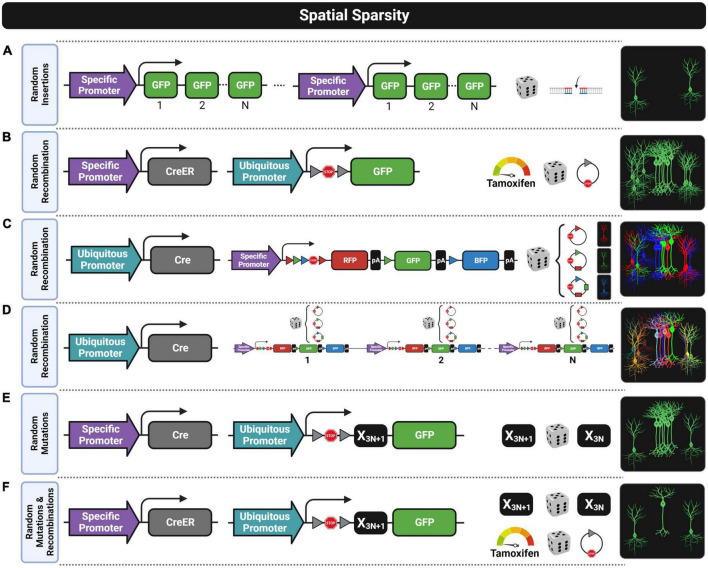
Fluorescent transgenic designs for spatially sparse labeling. Six approaches are shown by depicting from left to right the construct designs, the stochastic process for achieving spatial sparseness, and labeling patterns. **(A)** Random insertions of the construct into the genome can result in spatially sparse labeling that is usually biased since some cell subtypes within the complete population genetically defined by a specific promoter are usually unlabeled due to epigenetic silencing events. **(B)** Random recombination achieved with a low Tamoxifen dose when using the CreER/LoxP design results in a still dense labeling pattern when selecting a promoter with widespread expression. **(C)** Random recombinations in the Brainbow construct lead to three chromatic labelings (e.g., red, green, and blue) that do not provide enough chromatic discrimination for extracting individual morphologies. **(D)** Random recombination in a design having multiple copies of the Brainbow construct provides multiple chromatic labeling (e.g., red–green–blue combinations) suitable for chromatic discrimination, but the brightness of the fluorescent labeling is usually not good enough for morphological reconstructions. **(E)** Random mutations in the mononucleotide repeat frameshift (MORF) design provide spatially sparse labeling with some clustering patterns where morphological reconstruction might not be possible. **(F)** Random mutations and random recombinations in the MORF design combined with CreER/LoxP design usually provide a good enough spatially sparse labeling that is usually suitable for morphological reconstruction even using a promoter with widespread expression.

The first strategy for generating transgenic lines with spatial sparse labeling patterns was through inserting the transgene construct into a random locus of the genome, as shown in Figure 3A. As already mentioned, the chromatin state near the integration site highly defines the transgene expression (Josh Huang and Zeng, 2013) in such a way that transgene silencing events might occur in subsets of intended labeled cells that might share similar epigenetic profiles (Feng et al., 2000; Josh Huang and Zeng, 2013). This phenomenon is known as position effect variegation, and for this reason, the specific degree of spatial sparsity achieved in each of these transgenic lines highly depends on the specific insertion site of the construct (Luo et al., 2008). For instance, the Thy1-eYFP-H line shows slightly spatially dense labeling patterns (Feng et al., 2000) while the Thy1-eGFP-M line shows (Feng et al., 2000) a spatially sparse expression that has been successfully used to obtain high-quality morphological reconstructions of pyramidal neurons (Guo et al., 2017; Wang and Xiong, 2021). However, these reconstructions might be biased since labeled cells might represent a subset of pyramidal neurons. In addition, the expression pattern can be potentially different when crossing these transgenic lines with different genetic backgrounds or disease mouse models, since differences in the epigenetic profile can change the transgene silencing pattern. Therefore, interpretation of the results should be done carefully.

Another alternative to obtain sparse labeling is using a very low dose of tamoxifen in CreER drivers, which leads to stochastic recombination patterns when crossed with a Cre-inducible reporter (Figure 3B) resulting in a spatially sparse expression of the transgene (Ishino et al., 2017; Harris et al., 2019; Peng et al., 2020, 2021; Veldman et al., 2020). In this case, it is likely that the subset of labeled cells is unbiased since the probability for recombination seems to be equal for all cells. Unfortunately, a low dose of tamoxifen is not able to provide enough spatial sparsity when the promoter of the CreER driver has a widespread expression (e.g., Camk2a-CreER). In fact, morphological reconstructions are only possible with this procedure when using promoters that by default have an already moderate spatially sparse expression.

Another strategy, known as Brainbow, achieves spatial sparsity through color multiplexing by labeling each cell stochastically with a different color (Livet et al., 2007; Cai et al., 2013; Loulier et al., 2014), as shown in Figures 3C,D. The transgenic construct of these models usually has three sequences of different FPs flanked by pairs of lox variants that are mutually incompatible (e.g., loxP, lox2272, or loxN). The key feature of this design is that all possible recombination events have the same probability of occurring in the presence of Cre, so that only one fluorescent protein will be stochastically selected to be expressed (Figure 3C). Thus, one copy of this construct can only label one cell with one of the pure colors given by the limited FPs available in the sequence. Having more colors to label neurons is important to improve color discrimination between cells, especially when extracting anatomical features. For this reason, the number of colors is usually increased in these models by introducing into the genome multiple copies of the Brainbow construct (Figure 3D). As stochastic recombination events occur independently in each copy, cells can have different expression levels of all available FPs, making it possible to combine the colors for cell labeling. However, the number of copies should not be increased above an optimal threshold, as otherwise, color discrimination will be reduced (Sakaguchi et al., 2018). Unfortunately, this optimal number of copies is insufficient to drive a strong expression of FPs, and the signal intensity in Brainbow models is not bright enough for some applications. This limitation was faced by a proof-of-concept approach named Tetbow where the Tet-Off system was applied to enhance the intensity of the fluorescent signal without losing color discrimination (Sakaguchi et al., 2018). Some relevant improvements in this approach were that dendritic spines and axonal boutons are clearly visible by only using the native fluorescent signal while in Brainbow systems antibody staining against the FPs is required to enhance the signal for visualizing fine structures (Cai et al., 2013).

A clever proof-of-concept work (Lu and Yang, 2017) explored a pioneering strategy for sparse labeling of brain cells by placing multiple mononucleotide repeats not divisible by three (3N + 1, e.g., 22 repeats) before the transgene sequence that encodes the FP (see Figure 3E). Under standard conditions, this leads to a reading frameshift which impedes the correct translation of the reporter protein. However, during DNA replication or repair events, stochastic frameshift mutations, more likely to occur in mononucleotide repeats, can result in the correction of the initial constitutive frameshift (3N + 1, e.g., from 22 repeats to 21, 18, 15, 12, 9, 6, or 3) enabling the translation of the fluorescent protein. This strategy labels about 1% of the total population of cells expressing the D1 dopamine receptor (Lu and Yang, 2017). However, as the authors pointed out, the strength of the fluorescent signal was not good enough for visualizing full morphologies, especially thinner distal axons. For this reason, this clever strategy was applied to the TIGRE-TRE-2.0 reporter design (Daigle et al., 2018) resulting in a new Cre-inducible reporter line (Ai166) that provides both strong and spatially sparse expression (Peng et al., 2020, 2021; Veldman et al., 2020). This mononucleotide repeat frameshift (MORF) design provides enough sparsity for full morphology reconstruction when crossing a MORF reporter with any Cre driver line regulated by spatially sparse promoters, but sparsity is still not good enough when using promoters with a dense expression such as Camk2a. In those last cases, crossing the MORF reporter with a CreER driver line can lead to a suitable spatially sparse labeling pattern by administrating a very low dose of tamoxifen (Veldman et al., 2020), as shown in Figure 3F.

The cells labeled with the MORF system might, however, be also biased since the mitotic mutations that correct the frameshift in a particular cell will be inherited by its progeny. In some cases, the fluorescent progeny can create local clusters (Lu and Yang, 2017) that make difficult morphological reconstructions (Figure 3E), while in others, clustering might be alleviated by neurodevelopment mechanisms such as cell migration. However, in both cases, fluorescent cells from the same progenitor might be too similar to capture the full diversity of a particular cell type, so that spatial sparsity and morphological diversity are better achieved by postmitotic mutations restricted to correct the frameshift in a single cell-type basis. Alternatively, using a CreER driver with a MORF reporter and a very low dose of tamoxifen adds an extra source of stochasticity (Figure 3F).

## Timing of expression

### Inducible systems

The temporal control of expression of a fluorescent protein will depend on the selected promoter, and this can be problematic in many applications. For instance, some cells transiently express a gene during developmental stages but not in adult. This is the case of a subset of cells that only express the dopamine transporter gene (Slc6a3) prenatally (Madisen et al., 2010) so that studying the dopaminergic system in adults requires to activate fluorescent Slc6a3 expression postnatally. This can also be useful to bypass embryonic lethality or premature death when using some promoters that are active during neurodevelopment (Daigle et al., 2018). Likewise, as transgene overexpression can lead to potential side effects on animals (Quina et al., 2017; Daigle et al., 2018), activating the fluorescent protein expression just after behavioral tests can minimize potential confounding factors. This temporal control requires a genetic inducible system for switching on the expression of the fluorescent protein at the desired time point.

The most popular inducible tool is based on the CreER system (Gossen and Bujard, 1992; Feil et al., 1997) where Cre is fused to an estrogen receptor (ER) that does not allow Cre entering into the nucleus. The system is activated upon tamoxifen (TMX) administration or to its principal active form, 4-hydroxytamoxifen (4-OHT), which enables the translocation of CreER into the nucleus, which in turn can recombine the LoxP sites. In transgenic designs, a CreER driver (e.g., Thy1-CreER) is commonly crossed with a Cre-dependent reporter (e.g., CAG-LSL-GFP) in such a way that the LSL sequence preceding a fluorescent transgene is irreversibly removed *via* Cre-mediated excision once TMX is administered. Therefore, the expression can be switched on but not switched off, making this system useful for tagging subpopulation of neurons whose identity can be defined based on different timings of expression of precursor markers during developmental and perinatal periods. For instance, the first approach for labeling cortical chandelier cells (ChCs) was based on a neuronal precursor gene (Nkx2.1) which requires generating a double transgenic model (Nkx2.1-CreER;Ai14) where the transgene expression must be induced at P0–P1 through TMX administration for correct labeling (Taniguchi et al., 2013; Ishino et al., 2017). More recently, transcriptional studies found a postmitotic gene marker (Unc5b) for labeling ChCs (Paul et al., 2017; Bakken et al., 2021) that leads again to the development of another inducible driver line (Unc5b-CreER) that advantageously allows the induction in adult mice (Dudok et al., 2021). In this case, the induction is required to avoid labeling other subpopulations of cells that can also express this gene during development. Moreover, the CreER system has been widely used in fate mapping studies where the labeling is induced at different embryonic states with different progenitor markers to track and characterize subpopulations of neurons (Miyoshi et al., 2010; Taniguchi et al., 2011; Taniguchi, 2014; Lodato and Arlotta, 2015; Matho et al., 2021). The use of immediate early genes (IEGs) to generate inducible CreER driver lines (e.g., cFos-CreER or Arc-CreER) also provides a way to label those neurons that were especially activated by a specific sensory stimuli or experience (Guenthner et al., 2013; Leake et al., 2021; Roy et al., 2022) to mainly study sensory coding, memory, and valence encoding (DeNardo and Luo, 2017). In those cases, an optimal induction can be achieved by an injection of 4-OHT short before (approx. 1 h) starting the experimental condition, and mice should be sacrificed when fluorescent protein expression reaches detectable levels, typically after some days, for analysis (Guenthner et al., 2013). Otherwise, neurons not related to the targeted event will be tagged since transgene expression is irreversible once induced when using the CreER system. One common drawback to all these applications is the stochastic leakage of CreER into the nucleus in the absence of TMX which leads to a basal activity of recombination. Although this leakage issue was reduced with the second generation of the CreER system (CreERT2), fluorescence may be detectable in some cells before TMX administration (Álvarez-Aznar et al., 2020). In those cases, basal fluorescence should also be evaluated in control samples with no TMX injection. Interestingly, there are Cre-inducible reporter lines with different degrees of basal activity depending on the distance between pairs of LoxP sites flanking an STOP cassette (Álvarez-Aznar et al., 2020). As this length affects the recombination efficiency, shorter distances lead to reporter lines with high basal activity while larger ones lead to reporter lines with lower basal activity.

Other inducible genetic tools have been developed based on different SSR systems such as the FlpER (Hunter et al., 2005) or the DreER (He et al., 2017; Han et al., 2021). Nonetheless, the CreER system is still the most used as it is the one with the highest recombination efficiency (Dymecki and Kim, 2007; Han et al., 2021), which was further improved with the self-cleaved CreER (sCreER) variant (Tian et al., 2020). Another approach is splitting a recombinant protein into two inactive fragments where each is fused to two complementary chemical-inducible heterodimerization (CIHD) domains. The most popular is the dimerized Cre (Di-Cre) system (Jullien et al., 2003, 2007) where the two Cre fragments are recomposed into its constitutively active form after the administration of a specific heterodimerizer (i.e., rapalog). Based on this idea, a battery of new inducible systems was recently developed (Weinberg et al., 2019) using both different recombinant proteins and chemical inducers. The key advantage of these dimerized-inducible tools is their low basal activity, rapid induction, and high induced activity (Weinberg et al., 2019; Tian and Zhou, 2021), making them a good alternative to systems suffering from higher basal levels as those based on recombinant proteins fused to an ER element. Despite the advantages, these promising chemical-inducible tools have not yet been applied to develop fluorescent transgenic mice for whole-brain applications. One common limitation shared by chemical-inducible systems is the course time control to trigger the fluorescent protein expression. For instance, the recombination of LoxP sites takes at least 1 day after TMX administration in transgenic mice based on the CreER system (Zervas et al., 2004; Miyoshi et al., 2010). On the other hand, similar light-inducible systems have been recently developed (Lee et al., 2017; Weinberg et al., 2019; Tian and Zhou, 2021) to provide fine temporal control but at the cost of constraining the inducible fluorescent expression to a small region of the brain. Therefore, whole-brain expression is not achievable (yet) in mouse models using light-inducible strategies (Tian and Zhou, 2021), being only compatible with animal models that are already optical transparent, such as zebrafish embryos.

### Inducible and reversible systems

The tight control over the expression time window of the fluorescent protein is also essential for enabling correlations between transitional physiological states of neurons at different temporal scales and the events that triggered them. For instance, the activity of neurons can be interrogated with many anatomical markers (Hoffman, 2020) such as IEGs to precisely tag only those cells that were active during a particular event. Therefore, the expression of the fluorescent protein driven by an IEG (e.g., cFos or Arc) should ideally be switched on at the start and switched off at the end of the event of interest to avoid tagging neurons associated with past or future events. For this purpose, a genetic switch is required that ideally should rapidly achieve high expression when switched on and no basal expression when switched off (Jonkers and Berns, 2002).

There are mainly two genetic switches both based on the tetracycline (Tet) controlled system (Gossen and Bujard, 1992). One is the Tet-Off system where the use of a Tet-controlled transactivator (tTA) activates a Tet-responsive promoter (pTRE) for driving the expression of a downstream transgene that can be switched off by administering Tet or its more stable derivative form, doxycycline (Dox). The other is the Tet-On system where the use of a reverse tTA (rtTA) requires the administration of Tet/Dox to activate the pTRE for switching on the transgene expression. These Tet systems have some drawbacks. First, several side effects associated with the administrations of Tet/Dox are reported to affect the results in some experimental designs (Wüst et al., 2020). Second, tTA could be neurotoxic when expressed at high levels (Han et al., 2012; Daigle et al., 2018). Third, the TRE promoter when not activated for prolonged periods tends to be epigenetically silenced, especially in neurons (Zhu et al., 2007). The silencing seems to be more pronounced in the Tet-On system (rtTA) than in the Tet-Off (tTA) system, which might explain the lack of transgenic driver lines based on the Tet-On system for tagging neurons. Nonetheless, such epigenetic silencing is significantly improved when integrating the TRE promoter into the TIGRE locus (Zeng et al., 2008; Tasic et al., 2012) and by adding insulator elements flanking the construct (Madisen et al., 2015; Daigle et al., 2018). Interestingly, these silencing issues were only reported when using transgenic mice where the TRE promoter is integrated into the genome but not when using recombinant adeno-associated virus (rAAV) approaches where the virus remains in an episomal state, and hence, the construct is not integrated into the genome (Zhu et al., 2007). However, tTA drivers controlled by IEG promoters (e.g., cFos-tTA or Arc-tTA) have been extensively crossed with TRE reporter lines for whole-brain labeling (Reijmers et al., 2007; Garner et al., 2012; Tayler et al., 2013) or infected with TRE-dependent rAAV for regions-specific labeling (Liu et al., 2012; Roy et al., 2016, 2022; Choi et al., 2018). These designs allow to specifically tag neurons that were active within a temporal window constrained *via* Tet/Dox administration that enables, among others, studying memory within the theoretical framework of engram cells (Josselyn and Tonegawa, 2020). Despite these advances, there is still some room for improvements. For instance, there is no transgenic tool at this moment for permanently tagging two different populations of neurons that were active during two different behavioral tasks at different time windows (Guenthner et al., 2013). The double temporal labeling for whole-brain approaches usually requires combining transgenic lines for labeling neurons related with the first event while a second event can only be tagged short after mice are sacrificed with whole-brain immunostaining approaches (Yun et al., n.d.; Park et al., 2019). For this reason, a protocol is still missing for permanently double tagging those neurons that were active during a training event and a recall event (i.e., engram cells) for electrophysiological studies. Other alternative of using inducible systems for tagging events in constrained time windows is the use of transgenic mice based on destabilized fluorescent proteins (Reijmers et al., 2007; Kim and Cho, 2020; Meenakshi et al., 2021), such as the 2-h half-life GFP (shGFP).

## Subcellular localization

**FIGURE 4:**
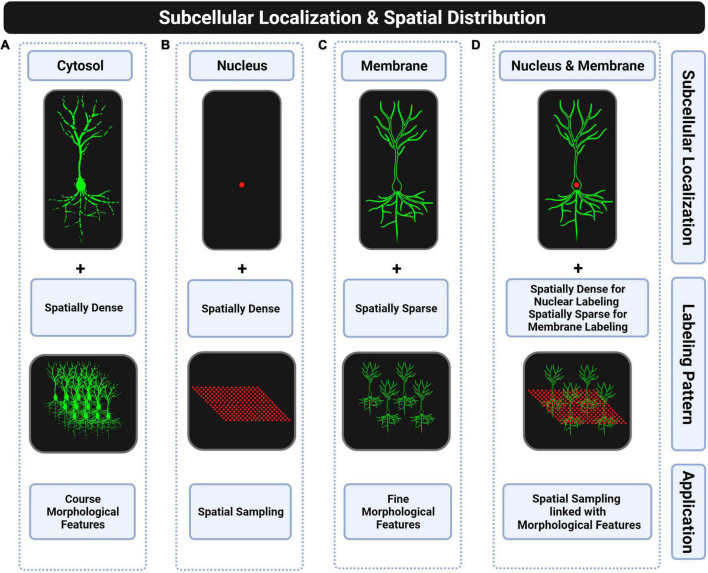
Subcellular locations of fluorescent proteins for whole-brain applications. **(A)** Cytosolic targeting with spatially dense labeling is suitable for extracting coarse morphological features such as dendritic or axonal density of a particular cell type. Discontinuities of the fluorescent signal in distal thin processes and too dense labeling make this approach unsuitable for morphological reconstructions. **(B)** Nuclear targeting with spatially dense labeling is suitable for counting complete cell-type populations. **(C)** Plasma membrane targeting with spatially sparse labeling is suitable for full morphological reconstructions. **(D)** Combining the two previous strategies is suitable for linking soma spatial distribution with morphological features of single neurons. However, this strategy has not been fully explored due to the lack of transgenic mice that combine both labels.

The fluorescent protein in transgenic mouse models is by default expressed in the cytosol and distributed across the cytoplasm by free diffusion (Van Den Pol and Ghosh, 1998; Yamaguchi et al., 2000). However, cell counting in whole-brain approaches with automated algorithms is challenging when using default cytoplasmic location of the fluorescent signal (Newmaster et al., 2021; Refaeli et al., 2021; Tyson and Margrie, 2022) mainly due to two reasons. First, light signals of neighboring cells in dense-labeled populations are usually merged due to light scattering making it impossible to discern single cells. Second, the huge diversity of shapes, sizes, intensities, backgrounds, and noise levels of labeled cells impedes automated cell detection (Tyson and Margrie, 2022). Instead, when using a nuclear labeling approach, there is a much wider space with no fluorescent signal in between nearby neurons that enables single-cell detections (see Figure 4B). Also, the shape and size of the nucleus are more stable across cells. The most commonly used fusion proteins to target the fluorescent protein into the nucleus are histone 2A (H2A), histone 2B (H2B), and the nuclear localization signal (nls) (Fraser et al., 2005; Jiang et al., 2008; Keller et al., 2008; Abe and Fujimori, 2013). In the context of transgenic mouse models, Cre-inducible reporter lines with nuclear labeling were generated based on H2B (Peron et al., 2015; He et al., 2016; Kim et al., 2017; Matho et al., 2021), nls (Daigle et al., 2018), and SUN1 which is a nuclear membrane protein (Mo et al., 2015; Fernandez-Albert et al., 2019). These lines allow studying the spatial distribution of particular cell types in whole-brain images (Kim et al., 2017), and delineating brain regions (Trotter et al., n.d.; Quina et al., 2020; Yao et al., 2020; Zhang G. W. et al., 2021) used to refine mouse brain atlases (Chon et al., 2019). When crossed with a pan-neuronal Cre driver, they can provide landmarks in whole-brain images where the fluorescent background signal is not enough to reveal the boundaries between brain regions. These Cre-inducible nuclear reporter lines such as the Ai75 reporter line (Daigle et al., 2018) can also be seen as an alternative to using nuclear dyes (e.g., propidium iodide) or antibodies (e.g., NeuN) for similar purposes, especially in cleared whole-brain applications. However, some deleterious effects were found at least in one reporter line based on the nls fusion protein (Ai75) such as hindlimb weakness (Quina et al., 2017), progressive decrease of fluorescent protein expression until fluorescence is lost (Daigle et al., 2018), and neurotoxic effects (Gasparini et al., 2019). Transcriptional and behavioral deficits were also found in nuclear reporter lines based on the H2B fusion protein due to an aberrant organization of the three-dimensional chromatin structure (Ito et al., 2014).

Cytoplasmic labeling does also not allow full morphological reconstructions of neurons since fluorescent signals present discontinuities in thinner distal processes (see Figure 4A). The complete labeling of neurons is only possible when targeting the fluorescent protein to the plasma membrane (see Figure 4C) by using fusion protein strategies (Muzumdar et al., 2007; Lindberg et al., 2013). Conversely, cytoplasmic labeling was proposed to be more suitable for Brainbow approaches (Sakaguchi et al., 2018; Abdeladim et al., 2019). Therefore, based on the literature, we could conclude that membrane labeling is preferred for conducting full morphological reconstructions of single neurons in monochromatic applications while cytoplasmic labeling results are usually selected for partial reconstructions in multi-chromatic applications as in Brainbow-based transgenics. There are also Cre-inducible reporter lines to extract the morphology of genetically defined cell types through a spatially sparse labeling strategy and by targeting the fluorescent protein to the plasma membrane, as shown in Figure 4C, such as the Ai166 reporter line (Veldman et al., 2020).

In fact, there is no all-in-one reporter line yet to extract at the same time the spatial distribution of complete populations of specific cell types and full morphological reconstructions (Figure 4D), which would help studying relationships between spatial distribution of somas and fine morphological features of neurons for revealing brain design principles such as spatial regularity of somas or spatial uniform coverage of dendritic arbors (Field and Chichilnisky, 2007; Reese and Keeley, 2015).

## Best models for tracing connectivity

While individual synapses cannot be faithfully measured at the whole mouse brain scale with optical microscopy, recently developed transgenic mouse lines provide interesting tools for cellular-scale descriptions of connectivity at the population level.

Traditionally, anterograde and retrograde tracer injections have been used to study axonal projections in the mouse brain, providing the first approximations of connectivity (Oh et al., 2014). However, the fine details of network connections at the cellular scale (those mainly altered in brain pathologies) cannot be explored. In the last two decades, genetically engineered rabies viruses and AAVs allow measuring monosynaptically connected neurons. The original work of Wickersham et al. (2007) described the induced expression of a modified SAD-B19 rabies virus genome replacing the coding sequence for the rabies glycoprotein (required for transsynaptic infection) with GFP and an avian virus envelope protein (EnvA). When this virus is injected into a mammalian brain, it does not infect any cell because there are no membrane receptors for EnvA. This allows to selectively label neuronal types expressing the EnvA receptor (TVA). By additionally deleting the gene of the viral glycoprotein (B19G, G-deleted rabies virus; RVdG), the authors also engineered transsynaptic transmission, constraining its expression only to infected cells. Only cells expressing the glycoprotein are able to transmit the RVdG material to input neurons. By adding fluorescent proteins, starter and presynaptic neurons can be labeled with different colors. Interestingly, as the glycoprotein is only expressed in the injection region, only monosynaptic neighbors will be infected, allowing for quantification of connectivity at the cellular scale. Even though the original glycoprotein variants were not able to label all presynaptic neurons, a codon-optimized version of a chimeric glycoprotein consisting of the transmembrane/cytoplasmic domain of B19G and the extracellular domain of rabies Pasteur virus strain glycoprotein led to improving the efficiency of transsynaptic labeling (Kim et al., 2016). However, rabies viral vectors imply neurotoxicity soon after injection (Wickersham et al., 2007; Osakada et al., 2011). This has been addressed by developing a self-inactivating RVdG (SiR; Ciabatti et al., 2017), allowing lifelong labeling of rabies-infected cells. By substituting the glycoprotein with a chimeric envelope protein of the vesicular stomatitis virus glycoprotein (VSV-G), the technique was adapted to allow anterograde tracing (Beier et al., 2011). To ensure monosynaptic labeling, VSV-G was supplied in trans (Beier et al., 2013). Similarly, thymidine kinase complementation of the herpes simplex virus (HSV) also allows anterograde monosynaptic tracing (Zeng et al., 2017). Still, given the non-toxic, persistent expression of AAVs, they have been widely used for studying efferent projections (Nassi et al., 2015) and have shown anterograde transsynaptic spread properties (mainly AAV1, Zingg et al., 2017, 2020), contributing substantially to the mapping of network connectivity (Suarez et al., 2018; Beltramo and Scanziani, 2019; Trouche et al., 2019; Fujita et al., 2020; Zingg et al., 2022).

A variant of rabies retrograde tracing technique has successfully been used in large-scale studies of mouse cortical connectivity (Yao S. et al., 2021). Similarly, full morphology reconstruction studies hold promise for describing the topology of whole-brain circuits at the cellular scale (Peng et al., 2021). Another successful approach to map single neuron projections has been the use of viral injection of random “barcode” RNA sequences (Kebschull et al., 2016), allowing to identify unique projections from thousands or millions of cells in putative projection areas. Along with the quantification of landmarks of synaptic connectivity (Jiang et al., 2022), these tools will allow a comprehensive mapping of mammal brain connectivity based on light microscopy. However, further work is needed to combine these promising approaches with the molecular, morphological, and functional classification of brain cells and their use in disease models.

These tracing connectivity approaches enable to label structural connected neurons but not functional connected ones. When using retrograde tracers, it is possible to know which upstream neurons are structurally connected to a given neuronal population, but we cannot know which of those upstream neurons are exciting the neuronal population of interest. Likewise, a similar approach using anterograde tracers encounters the same problem. We cannot tell which downstream neurons were activated by the presynaptic element of interest. In fact, an active dependent tracing tool at a single neuron resolution is still missing.

## Combinatory strategies

### Intersectional targeting for single labeling

For a long time, neuroscientists have been trying to classify neurons into cell types based on several criteria such as developmental origin, precise spatial location (e.g., brain region or laminar position), morphology, electrophysiological properties, connectivity pattern (e.g., axonal projecting regions and synaptic targeting of specific subcellular compartments), neurotransmitter expression, transcriptomic profile, and/or computational role in circuits, among others. Despite the efforts, a systematic taxonomy to precisely name functional cell types is still an open issue (DeFelipe et al., 2013; Josh Huang and Zeng, 2013; Lodato and Arlotta, 2015; Zeng and Sanes, 2017; Yao Z. et al., 2021) that is limiting our capacities to disentangle brain circuits. These putative neuronal classes can be correlated with gene expression profiles where more than a single gene is commonly required to unambiguously constrain their identities. As a result, fluorescent transgenic approaches were developed to refine cell-type identities by intersecting combinations of expressed genes. In the simplest case, cells expressing two specific genes (e.g., gene A and gene B) can be particularly labeled through two different intersectional designs where both require generating triple transgenics by crossing together two drivers and a reporter line. As will be described, one is based on combining different site-specific DNA recombinase systems and the other on mixing a recombinase with a transactivator system.

**FIGURE 5:**
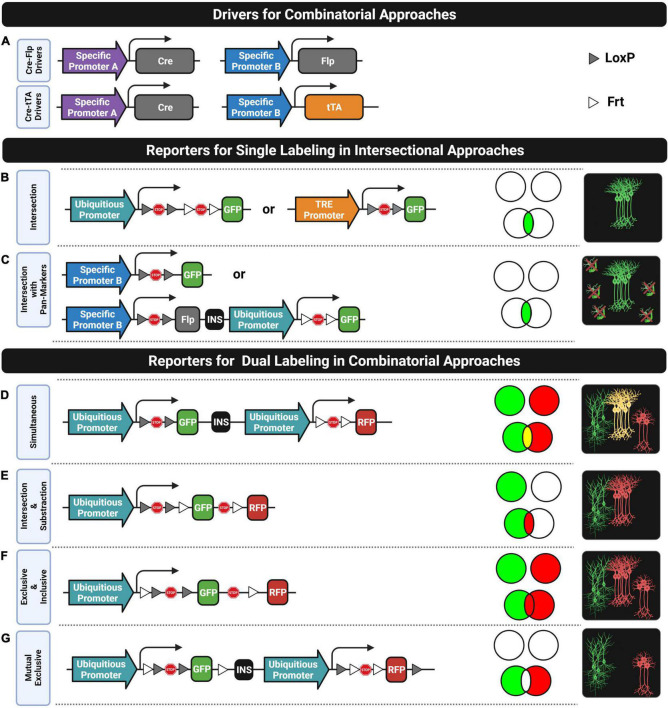
Combinatorial strategies. **(A)** Two driver combinations (Cre-Flp and Cre-tTA) widely used for labeling cell subtypes based on two genes. **(B,C)** Reporter designs compatible with single labeling in intersectional approaches that require generating triple transgenic **(B)** or double transgenic **(C)** mice. **(D–G)** Reporter designs compatible with dual labeling in combinatorial approaches. Venn diagrams indicate the labeling subsets for each reporter design in two situations: when the two genes are mutually exclusive in all cells (top) and when their expression overlaps in some subset of cells (down). Rightmost diagrams represent the labeling pattern only in the overlapping gene condition.

As shown in Figure 5B, the first design involves using two driver lines where the expression of different recombinases (e.g., Cre and Flp) is driven by different gene-specific promoters (e.g., promoter A and promoter B) and an intersectional reporter line consisting of a ubiquitous promoter followed by two STOP cassettes flanked by the corresponding recombination sites (e.g., a LoxP-STOP-LoxP cassette [LSL] and an Frt-STOP-Frt cassette [FSF]) that prevent the downstream expression of a fluorescent protein. In this intersectional triple transgenics, only those cells expressing both genes will be labeled. The most popular implementation of an intersectional strategy consists of crossing together a Cre driver, a Flp driver, and a Cre-Flp-dependent intersectional reporter line that has resulted in the development of several intersectional reporter lines such as the RCE-dual (Miyoshi et al., 2010) and Ai65D (Madisen et al., 2015) for cytoplasmic labeling, and the HG-dual (He et al., 2016) for nuclear labeling. These reporters have been extensively used for improving the identities of both neural progenitors in fate mapping studies (Miyoshi et al., 2010; He et al., 2016) and postmitotic neurons in diverse applications where accurate labeling of a particular subtype is critical. For instance, labeling inhibitory interneurons can be difficult without using intersectional approaches. In most cases, using solely one gene marker of a particular interneuron subtype can also label some excitatory neurons. For this reason, an additional pan-GABAergic marker (e.g., Dlx5/6 or Slc32a1) is commonly required for labeling exclusively the intended inhibitory interneuron subtype. As a result, intersectional Cre-Flp triple transgenic lines such as the CCK-Cre;Dlx5/6-Flp;RCE-dual (Taniguchi et al., 2011), Slc32a1-Cre;Pvalb-Flpo;Ai65 (Madisen et al., 2015) and Cdh6-CreER;Dlx5/6-Flp;Ai65 (Ishino et al., 2017) were generated to respectively, label cholecystokinin (CCK) expressing interneurons, parvalbumin (PV) expressing interneurons and chandelier cells from the hippocampus. Moreover, the intersection between pairs of classical interneuron markers is also fundamental to study the diversity within these groups. In fact, this approach was successfully used for targeting neuronal subtypes that were already named for a long time based mainly on their morphology, electrophysiological, and connectivity profiles. For instance, the use of Cre-Flp intersectional triple transgenics enables to split vasoactive intestinal peptide (VIP) expressing interneurons into two subpopulations (He et al., 2016): the ones that co-express calretinin (CR) for labeling bipolar interneuron-selective interneurons (CR-Cre;VIP-Flp;Ai65) and the ones that co-express CKK for labeling small basket cells (CCK-Cre;VIP-Flp;Ai65). Likewise, somatostatin (SST)-expressing interneurons can also be split into two subpopulations (He et al., 2016): the ones that co-express CR for labeling Martinotti cells (CR-Cre;SST-Flp;Ai65) and the ones that co-express neuronal nitric oxide synthase (nNOS) for labeling long-range inhibitory neurons (nNOS-CreER;SST-Flp;Ai65). More recently, the intersectional Cre-Flp strategy was also applied for labeling two subpopulations of serotonergic neurons that differ in their axonal projecting targets (Ren et al., 2019) and for whole-brain mapping neurons that co-express different neurotransmitters (Xu et al., 2022) such as vesicular glutamate and gamma-aminobutyric acid (GABA) transporters (VGLUT and VGAT, respectively).

There is another intersectional design, as shown in Figure 5B, that involves crossing together a driver line expressing a recombinase (e.g., Cre) under one specific gene promoter, a second driver line expressing a transactivator (e.g., tTA or rtTA) under the control of another gene-specific promoter, and an intersectional reporter line consisting of a transactivator-responsive promoter (e.g., pTRE) followed by a STOP cassette flanked by the pertinent recombination sites (e.g., LSL) that prevents the fluorescent protein expression. This expression will only take place in those cells expressing both genes and if the transactivator system is switched on (e.g., in the absence of Tet/Dox when using tTA or in the presence of Tet/Dox when using the rtTA system). The generation of a Cre-tTA intersectional triple transgenic is the most common implementation of this design which involves crossing a Cre driver, a tTA driver (second row, Figure 5A), and a Cre-tTA-dependent reporter (second drawing, Figure 5B) as the Ai62 and Ai82 (Madisen et al., 2015). Unfortunately, despite the strong transgene expression achieved by this design, only few tTA driver lines were developed, but this is changing. Recently, new tTA drivers were generated and characterized for intersectional applications that selectively target neuron populations in different subregions of the cortex, hippocampus, and amygdala (Xu et al., 2022). Moreover, Cre-tTA intersectional triple transgenics were not applied yet for whole-brain mapping specific subtypes of neurons (e.g., using a PV-Cre driver) that were particularly active during a behavioral task (e.g., using a IEG-tTA driver such as a cFos-tTA or Arc-tTA driver). Instead, the cell identity of active neurons is commonly interrogated through immunostaining techniques applied to thin sections of brain tissue (Stefanelli et al., 2016). On the other hand, cell specificity can also be improved based on the temporal coincidence of multiple gene expressions by combining intersectional inducible systems (e.g., CreER, FlpER, and DreER).

There is also an intersectional design that only requires a double transgenic instead of a triple one. It consists of a Cre driver line which provides the specificity of one gene (e.g., promoter A) and a Cre-dependent reporter line where the fluorescent expression is driven by another gene-specific promoter (e.g., promoter B) instead of a ubiquitous one (see the first row of Figure 5C). This strategy is usually applied in those cases where a particular gene is selectively expressed in a subpopulation of neurons of interest but also in non-neuronal cells, such as in glial or vascular cells. The intersection of this gene with a pan-neuronal gene (e.g., SNAP-25 or hSYN-1) will constrain the labeling to only neurons. For this purpose, the promoter of the Cre-dependent reporter line is selected to be a pan-neuronal one (Madisen et al., 2015). A limitation of this system is that the strength of the pan-neuronal promoter for driving the fluorescent expression might not be strong enough for some applications. This might be addressed by slightly modifying the reporter line, as is proposed in the second row of Figure 5C.

### Combinatorial targeting for multiple labeling

Labeling two or more populations of neurons with different fluorophores can be fundamental for many applications. There are at least three main ways for multiple genetic labeling: crossing reporters, multiple vector-based co-injections into fertilized oocytes, or crossing together driver and reporter lines. The first approach involves crossing several reporter lines where the expression of the fluorescent protein is directly driven by a specific promoter of a particular gene. For instance, neurons expressing different dopamine receptors in the striatum (Drd1a and Drd2) can be labeled by crossing two reporter lines expressing different fluorophores (Drd1a-tdTomato;Drd2-EGFP). This double reporter transgenic was generated for labeling the two populations of striatal medium spiny neurons (Shuen et al., 2008) that mediate the striatal direct “GO” and indirect “STOP” pathways in control decision-making behaviors. The approach of crossing reporters has two main drawbacks: incomplete labeling and inefficient crossing. First, labeling the complete population of cells expressing the targeted gene is difficult to achieve with this type of reporter lines where the epigenetic silencing of the transgene in some subpopulations of cells is quite common since they are commonly generated by inserting the construct into random locations of the genome (Feng et al., 2000; Josh Huang and Zeng, 2013). Second, labeling more than two populations of cells might involve crossing many heterozygotic transgenic lines where only a low percentage of the progeny will inherit all transgenes. For instance, in the case of having three heterozygotic transgenics, only 12.5% of the progeny will present the three transgenes. This inefficient crossing problem is popularly known as the “allele problem,” and the second strategy enumerated above for achieving multiple genetic labeling was, in fact, proposed for solving it. This solution is a protocol named “Prism” that consists of co-injecting multiple transgene constructs into the same fertilized mouse egg for directly generating a single multiple transgenic line without the need of crossings (Dougherty et al., 2012). In the first proof of concept, they were able to generate a triple transgenic line where different promoters of specific genes drive the expression of different fluorophores in neurons (Snap25), astrocytes (Aldh1L1), and oligodendrocytes (Mobp) to assess functional interactions between these cell types (Dougherty et al., 2012; Gaire et al., 2018). Nonetheless, the third listed solution (i.e., crossing drivers and reporters) is the most popular approach for multiple labeling for several reasons. Mainly, the increasing number of driver and reporter lines currently available provides multiple ways to combine them to find the crossing most suitable for a particular application. In addition, driver lines are commonly designed with knock-in-based strategies for recapitulating more faithfully the endogenous gene expression, and reporter lines are commonly designed with strong ubiquitous promoters for driving high expression levels of fluorescent proteins. Moreover, there are available many reporter lines designed to label different combinatorial subsets of the genes targeted by the driver lines, as is shown by the Venn diagrams in Figure 5.

There are Cre-Flp-dependent dual-reporter lines such as the Ai193 line (Zingg et al., 2022) for dual simultaneous labeling of cells expressing one gene with one fluorophore and cells expressing another gene with another color, while cells co-expressing both genes, if they do, will be labeled with both colors (see Figure 5D). Likewise, there are even triple-reporter designs such as the Ai213 line for simultaneously targeting cells expressing different combinatorial subsets of three genes (Graybuck et al., 2021). Strikingly, these reporter designs have not yet been used in many studies, despite their potential applications. On the other hand, intersection and subtraction (IS) reporter lines (Figure 5E) have been used extensively in many studies for labeling with two different fluorophores cells co-expressing two genes (intersection subset) and cells expressing one gene but not the other (subtraction subset). The first application that took advantage of the IS reporter design was intersectional fate mapping of neuronal progenitors (Awatramani et al., 2003; Branda and Dymecki, 2004; Farago et al., 2006; Joyner and Zervas, 2006; Dymecki and Kim, 2007; Matho et al., 2021). They were also applied to target inhibitory neurons with gene markers that unfortunately are also expressed in excitatory neurons by intersecting these genes with pan-GABAergic ones (Nguyen et al., 2020). Although the intersection can also be targeted with an intersectional reporter (see the previous subsection), an IS reporter also labels with a second color, those cells positive for the pan-GABAergic gene but negative for the target gene expressed by the subset of inhibitory neurons of interest. This allows us to know the proportion of a targeted cell subset in relation to a reference one. Another application using dual-IS reporters is to check whether a gene marker that labels a particular cell type co-expresses a particular gene or not. For instance, cortical chandelier cells (ChCs) which can be roughly defined with a single gene (Nkx2.1) can be divided into PV-positive and PV-negative subsets by mapping them with two different colors through the use of an IS reporter line (He et al., 2016). Moreover, there are also IS reporter lines for intersecting and subtracting up to three genes. For instance, labeling exclusively the noradrenergic neurons of the locus coeruleus (LC) already requires intersecting two genes (Dbh and En1). This means that exploring subpopulations of neurons within the LC requires at least an additional third gene marker. In one study, the neuropeptide galanin (Gal) was used as the third marker to visualize with two colors the subpopulation of Gal-positive cells (triple intersection) and Gal-negative cells (double intersection and subtraction) in the LC by using a triple-IS reporter line (Plummer et al., 2015).

Furthermore, many dual-reporter designs have been developed for labeling different combinatorial subsets based on two genes markers by slightly rearranging the places of recombinase sites and stop sequence, as is shown in Figure 5F. Most of them were used for many applications such as for understanding stem cell fate plasticity and tracing (Zhao and Zhou, 2019; Liu et al., 2020) but they were not exploited yet for neuroscience research. There are even unexplored dual-reporter designs that might be useful for some applications, such as the one proposed in Figure 5G for mapping two populations of cells where the cell subset that co-expresses the two genes is avoided.

## Author contributions

AA and LM-G wrote the manuscript. AA elaborated all the figures. MD oversaw the manuscript and edited it. AA, MD, and LM-G had the idea, wrote the manuscript, contributed to the article, and approved the submitted version.

## Footnotes

1
https://mice.jax.org/

2
https://www.mmrrc.org/

3
https://bacpacresources.org/
